# Seasonal Patterns of Nitrogen and Phosphorus Limitation in Four German Lakes and the Predictability of Limitation Status from Ambient Nutrient Concentrations

**DOI:** 10.1371/journal.pone.0096065

**Published:** 2014-04-22

**Authors:** Sebastian Kolzau, Claudia Wiedner, Jacqueline Rücker, Jan Köhler, Antje Köhler, Andrew M. Dolman

**Affiliations:** 1 Brandenburg University of Technology Cottbus - Senftenberg, Department of Freshwater Conservation, Bad Saarow, Germany; 2 Leibniz-Institute of Freshwater Ecology and Inland Fisheries, Berlin, Germany; 3 Berlin Senate - Department for Urban Development and the Environment, Berlin, Germany; University of Shiga Prefecture, Japan

## Abstract

To identify the seasonal pattern of nitrogen (N) and phosphorus (P) limitation of phytoplankton in four different lakes, biweekly experiments were conducted from the end of March to September 2011. Lake water samples were enriched with N, P or both nutrients and incubated under two different light intensities. Chlorophyll *a* fluorescence (Chla) was measured and a model selection procedure was used to assign bioassay outcomes to different limitation categories. N and P were both limiting at some point. For the shallow lakes there was a trend from P limitation in spring to N or light limitation later in the year, while the deep lake remained predominantly P limited. To determine the ability of in-lake N:P ratios to predict the relative strength of N vs. P limitation, three separate regression models were fit with the log-transformed ratio of Chla of the P and N treatments (Response ratio = RR) as the response variable and those of ambient total phosphorus:total nitrogen (TN:TP), dissolved inorganic nitrogen:soluble reactive phosphorus (DIN:SRP), TN:SRP and DIN:TP mass ratios as predictors. All four N:P ratios had significant positive relationships with RR, such that high N:P ratios were associated with P limitation and low N:P ratios with N limitation. The TN:TP and DIN:TP ratios performed better than the DIN:SRP and TN:SRP in terms of misclassification rate and the DIN:TP ratio had the highest *R^2^* value. Nitrogen limitation was predictable, frequent and persistent, suggesting that nitrogen reduction could play a role in water quality management. However, there is still uncertainty about the efficacy of N restriction to control populations of N_2_ fixing cyanobacteria.

## Introduction

Anthropogenic eutrophication is one of the biggest threats to freshwater ecosystems. Its consequences include changes in phytoplankton species composition and increases in biovolume that are accompanied by unpleasant odors, oxygen depletion, decreases in water transparency and a loss of biodiversity [1], [2]. There has been an extended debate over whether nitrogen (N) or phosphorus (P) is the nutrient that ultimately determines productivity in lakes [3]–[5]. Early work emphasized P as the main nutrient controlling phytoplankton biovolume in most lakes based on inferences from the stoichiometry of N and P in phytoplankton and the relative availability of these elements in nature [4]. This view was further reinforced by observation of the close statistical relationship between chlorophyll a and P concentration [6] and the results from early lake manipulation experiments [7]. However, subsequent nutrient addition experiments have found N to be just as often limiting as P [8], [9] and it is now clear that the ratio of N to P in lakes varies widely so that many have a deficit of N relative to P [10]. Some authors stated that N limitation can only be observed in short-term, small-scale experiments that may not be relevant to dynamic lake systems, and argued that P is the ultimate limiting nutrient over time due to N_2_ fixation by cyanobacteria [3], [11]. However Spivak et al [12] showed that the results from small-scale experiments can be applied to larger more natural systems and in fact there are cases where N limitation was observed in mesocosms and whole lake experiments [13]. Scott and McCarthy [14] even interpreted the results of Schindler et al. [3] as proof that N_2_ fixing cyanobacteria cannot fully compensate for nitrogen limitation, as the total N concentration and chlorophyll *a* concentration decreased after the N fertilization was stopped. Paterson et al. [15] however responded in a comment with 4 more years of data for the studied lake showing that the N_2_ fixation increased and the chlorophyll *a* concentration remained at a high level without N fertilization.

The variety of results suggests that rather than a single nutrient determining lake productivity, the limiting nutrient may vary with lake type, trophic status and season. For example, Downing and McCauley [10] showed that average N:P ratios decline with trophy, so that N limitation is more likely to occur in eutrophic lakes while most oligotrophic lakes are likely limited by P. Reynolds [16] indicated that in deeper lakes at higher altitudes, P sets the upper limit of phytoplankton biovolume, but that this is less likely to apply to smaller, shallower lakes at all altitudes. Morris and Lewis [17] found 5 of 8 lakes in the Colorado mountains where the limiting nutrient changed during the year. However studies that investigated limitation in multiple lakes differing in mixing type, and over whole growing seasons, are still rare.

Reducing nutrient inputs from sewage plants or agriculture is expensive [18], [19]; therefore it may be more cost and time effective to match reduction measures for specific nutrients to regions or phases when the nutrient in question is limiting and thus may have an immediate effect on water quality. The most commonly used method to identify the limiting nutrient is the enrichment bioassay, e.g. [20], in which different nutrients are added to separate water samples and the response of the phytoplankton is monitored. These experiments can be conducted on different temporal and spatial scales, ranging from bioassays in small bottles with duration of hours to a few days, e.g. [21], to whole lake manipulations that can run indefinitely, e.g. [7], [22]. Small scale experiments offer tight control over experimental conditions, like temperature and light intensity, but may exclude important processes operating in natural systems. With increasing size and duration, experiments more closely replicate natural systems and include processes such as nutrient fluxes at the water-sediment interface; however, this comes at a cost of reduced experimental control, smaller sample sizes [23] and a lack of replication. Furthermore, due to their ecological impact, whole lake experiments usually cannot be used to determine limiting nutrients in lakes. There are some downsides to identifying the limiting nutrient by experiments. Even small scale nutrient enrichment bioassays are time and cost intensive and cannot be repeated for large numbers of sites or over long periods. Therefore it would be useful to be able to predict the outcome of these experiments from in-lake nutrient concentrations, which are part of most monitoring sampling programs. Theories of predicting the limiting nutrient with the elemental composition of the phytoplankton or the composition of the water bear on the work of Redfield [24] who observed that on average phytoplankton assimilate C, N and P in the molar ratio of 106∶16∶1 (mass N:P ratio of about 7). This very generalized ratio has to be used carefully because it may vary with ecosystem and scale of analysis [25]. Morris and Lewis [17] tested nine indices to predict the limiting nutrient in Colorado mountain lakes and found the DIN:TP ratio to be the best predictor. Subsequent studies have similarly found DIN:TP to predict the limiting nutrient the best in boreal and alpine lakes [26] and the Baltic Sea [27].

The aim of this study was to compare the seasonal patterns of N and P limitation in four German lowland lakes, differing in mixing type, and to test which N:P ratio best predicted the limiting nutrient. Biweekly bioassays were conducted between the end of March and September 2011, in one deep-stratified, two shallow-polymictic and one riverine lake in the Berlin/Brandenburg lowlands. Each bioassay experiment then was classified into limitation categories by model selection. The seasonal pattern of limitation was compared with the seasonal dynamics of nutrients, available light and phytoplankton biovolume to identify drivers of the limiting factors. As quantitative measure of nutrient limitation a response ratio was calculated. With linear regression this response ratio was then used to test the predictive power of the dissolved inorganic nitrogen to soluble reactive phosphorus (DIN:SRP), total nitrogen to total phosphorus (TN:TP), TN:SRP and DIN:TP ratio. We found that the seasonal patterns of limitation differed between lakes of different mixing type and that the limiting nutrient could be predicted by DIN:TP and TN:TP ratio.

## Materials and Methods

### Ethic Statement

No permits or approvals were required for the field studies at Scharmützelsee (52.216°N, 14.024°E), Langer See (52.243°N, 13.786°E), Müggelsee (52.438°N, 13.645°E) or Untere Havel (52.449°N, 13.157°E). During the study no privately owned or protected land was accessed and the study did not involve endangered or protected species.

### Data Availability Statement

All data underlying the findings reported in this study can be found in the supporting information **Table S1–S3**.

### Study Sites, Nutrient Concentrations, Phytoplankton Biovolume and Light Availability

The nutrient and light limitation status of four lakes in the German states of Berlin and Brandenburg were studied from the end of March to September 2011: a deep stratified lake (Scharmützelsee = SCH), a very shallow polymictic lake (Langer See = LAN), a shallow temporarily stratified lake (Müggelsee = MUEG) and a shallow riverine lake (Untere Havel = UH). The main characteristics of the lakes are shown in Table 1; for more detailed information see Grüneberg et al. [28], Nixdorf and Deneke [29], Köhler et al. [30] and Knösche [31].

**Table 1:** Morphometric data, geographic coordinates, mean concentration of TN, TP and chlorophyll *a* for the lakes from the end of March to September 2011.

Lake	Mixis	A (km^2^)	Geographic coordinates	z_max_ (m)	z_mean_ (m)	TN (µg L^−1^)	TP (µg L^−1^)	Chla (µg L^−1^)
SCH	di	12.1	52.216°N, 14.024°E	29.5	8.9	594	26	15
LAN	poly	1.6	52.243°N, 13.786°E	3.8	2.1	836	61	61
MUEG	poly	7.5	52.438°N, 13.645°E	8.9	4.8	1278	84	31
UH	poly	11.7	52.449°N, 13.157°E	10.7	5.6	1491	103	24

A = lake area; z_max_ = maximum depth; TP = total phosphorus; Chla = chlorophyll *a*.

Water sampling was performed biweekly at the deepest point of SCH, in the southern main basin of UH, in the middle of LAN, and weekly at five stations spread across MUEG. For SCH and MUEG, subsamples were taken from the mixed part of the water column (i.e. the epilimnion during thermal stratification or the whole water column during mixing periods) at 1 m depth intervals with the volume taken at each depth proportional to the lake volume at that depth. For UH and LAN, equal volume subsamples were taken at 0.5 m depth intervals. Subsamples were mixed together and used for the following experiments and analyses. Concentrations of soluble reactive phosphorus (SRP), total phosphorus (TP), nitrate plus nitrite (NO_total_-N), ammonia (NH_4_-N) and total nitrogen (TN) were measured according to standard methods [32]. Herein we refer to the sum of NO_total_-N and NH_4_-N as dissolved inorganic nitrogen (DIN). Phytoplankton biovolume and species composition were estimated according to Utermöhl [33] using an inverted microscope. Secchi depth (z_SD_) and depth profiles of water temperature were measured. The mean photosynthetically active radiation (PAR) in the mixed upper part of the water column (I_mix_) was assumed to approximate the in situ light conditions for phytoplankton and was calculated according to Wiedner et al. [34]:
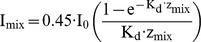
where z_mix_ is the mixing depth, K_d_ is the vertical attenuation coefficient and I_0_ is the mean global radiation for that specific calendar week. Global radiation data from the meteorological observatory in Lindenberg were used. For LAN and UH the mean depth, and for SCH and MUEG the depth of the epilimnion (from the surface to the point where the change in water temperature was greater than 1°C per meter), was used as mixing depth. When the epilimnion was deeper than the mean depth, the mean depth was used as mixing depth. The mean depth was determined with bathymetric maps drawn with sonar and GPS data. For SCH, LAN and UH the vertical attenuation coefficients were calculated from Secchi depth using an equation derived from long-term data of regional turbid lakes of different trophic states as given in Hilt et al. [35]:



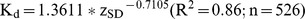



For MUEG the vertical attenuation coefficients were calculated according to Kirk [36]:
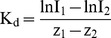
where I_1_ and I_2_ are the PAR at depth z_1_ and z_2_ respectively.

### Bioassays

**Figure 1:**
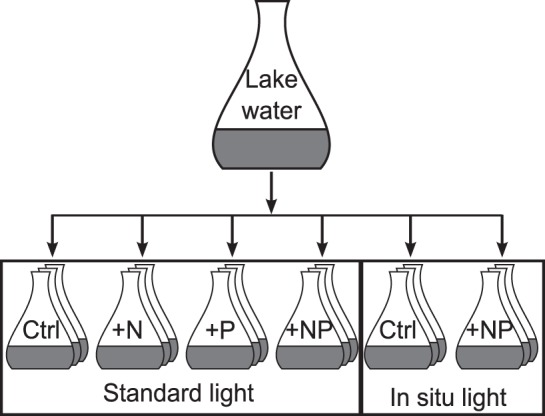
Experimental design of the bioassays. Treatments: Ctrl = control (no nutrient addition); +N = 250 µg N L^−1^ each of NaNO_3_ and (NH_4_)_2_SO_4_; +P = 200 µg P L^−1^ of KH_2_PO_4_; +NP = combined N+P addition; Standard light = 100 µmol photons m^−2^ s^−1^; In situ light  =  predicted I_mix._

Nutrient addition experiments (bioassays) were conducted every two weeks between the end of March and September 2011. The full experimental design (Figure 1) consisted of six treatments, four incubated under standard light conditions and two under in situ light conditions. In all cases, a control with no nutrient addition (Ctrl); an addition of 500 µg L^−1^ nitrogen in the form of 250 µg N L^−1^ (NH_4_)_2_SO_4_ and NaNO_3_ (+N); an addition of 200 µg L^−1^ phosphorus in the form KH_2_PO_4_ (+P) and the addition of both nutrients like in the single nutrient additions (+NP) were incubated under a standard light intensity (SL) of 100 µmol photons m^−2^ s^−1^. 100 µmol photons m^−2^ s^−1^ were chosen because at this light intensity we did not expect light to be limiting nor inhibiting. Before each experiment, and for each lake, the in situ I_mix_ was estimated (see above). When the in situ light conditions were expected to be below 75 µmol photons m^−2^ s^−1^, separate replicates of the control and +NP treatment were additionally incubated under the estimated in situ light conditions (ISL). Between 75 and 100 µmol photons m^−2^ s^−1^ we expected the effect on growth of the difference between SL and ISL treatments to be too small to be reliably detected and therefore decided to conduct ISL treatments only when I_mix_ was below 75 µmol photons m^−2^ s^−1^. Therefore SL and ISL treatments were both performed in 20 experiments and just the SL treatments were performed in 29 experiments. Osram Lumilux cool daylight fluorescent tubes were used as the light source.

All bioassays were started on the same day as sampling, with water from the same sample as that for the nutrient analyses. Larger zooplankton were removed from the water by prefiltering through a 200 µm gauze. For all treatments three replicates of 150 ml lake water were incubated, gently shaken in glass Erlenmeyer flasks in a growing chamber (KBW 400, Binder), for three days at the measured water temperature of the epilimnion (±2°C) under a 12 h: 12 h light: dark regime. The bottles were closed with cotton plugs to maintain air supply and bottle positions were switched daily to adjust for a light gradient in the growing chamber. The response of the phytoplankton was determined by measuring chlorophyll *a* concentration after three days with a fluorescence probe (FluoroProbe, bbe-Moldaenke).

### Limitation Categories

**Figure 2:**
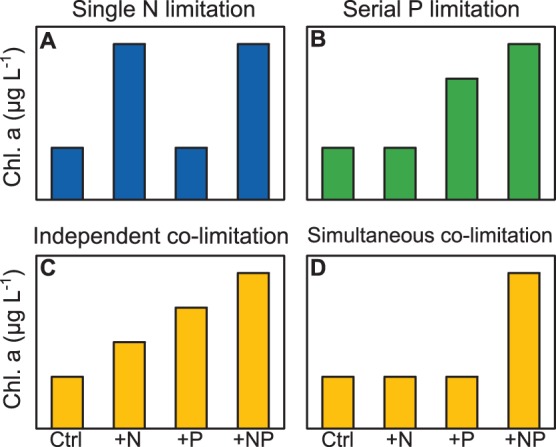
Example chlorophyll *a* response patterns. These patterns correspond to a subset of the nutrient limitation categories defined by Harpole et[37]. **A)** Single N limitation: a response to only one of the single treatments, in this example +N and the response to the +NP treatment is no different. **B)** Serial P limitation: a response to only one of the single nutrient treatments, in this example +P and a larger response to the +NP treatment. **C)** Independent co-limitation (primary P): a response to both single nutrient treatments with a larger response to +P and an even larger response to the +NP treatment; **D)** Simultaneous co-limitation: a response only to the +NP treatment.

The outcomes of the bioassays were classified according to the 8 nutrient limitation categories defined by Harpole et al. [37] and illustrated in Figure 2:

Single limitation (N or P): response to only one of the single nutrient treatments (+N or +P) and the response to the combined treatment (+NP) is no different (Figure 2A).
Serial limitation (N or P): response to only one of the single nutrient treatments (+N or +P) but a larger response to the +NP treatment (Figure 2B).
Independent co-limitation (primary N or P): response to both single nutrient treatments and a larger response to the +NP treatment; the single treatment with the larger response indicates the primary limiting nutrient (Figure 2C).
Simultaneous co-limitation: response only to the +NP treatment (Figure 2D).
No nutrient limitation: no response to any nutrient treatment (not shown).

**Figure 3:**
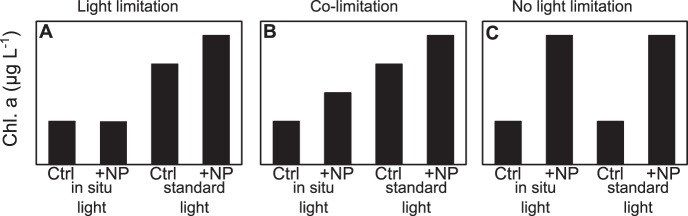
Possible light limitation patterns. **A)** Light limitation: a lower response to the Ctrl and +NP treatments when incubated under in situ light conditions and no difference between the Ctrl and +NP treatment when incubated with in situ light. **B)** Co light-nutrient limitation: the +NP treatment response is greater than the Ctrl for both standard and in situ light, but responses to Ctrl and +NP treatments are lower under in situ light than standard light. **C)** No light limitation: no difference between in the in situ and standard light incubation for either the Ctrl or +NP treatment.

In addition, three light limitation categories were distinguished:

Light limitation: a lower response to the Ctrl and +NP treatments when incubated under in situ light conditions compared to the response when incubated under standard light intensity and no difference between the Ctrl and +NP treatment when incubated with in situ light (Figure 3A).
Co-light-nutrient limitation: the +NP treatment response is greater than the Ctrl for both standard and in situ light, but responses to Ctrl and +NP treatments are lower under in situ light than standard light (Figure 3B)
No light limitation: no difference between the in situ and standard light incubation for either the Ctrl or +NP treatment (Figure 3C).

A model selection procedure was used to assign bioassay outcomes to one of the above categories in a similar manner to Andersen et al [38]. Nutrient and light limitation categorization were performed separately. For treatments incubated under standard light, a set of linear models were fit to each bioassay outcome where each model represents one of the nutrient limitation categories outlined above. The simplest model corresponds to a no-response classification and has a single parameter b_0_ representing the mean chlorophyll *a* (Chla) for all treatments:
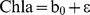



The model representing single N limitation has two parameters: one representing the mean chlorophyll *a* for all treatments where N was added 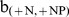 and one for all other treatments 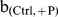:
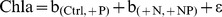



The most complex model has a separate parameter for all treatments:
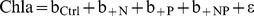



Akaike’s Information Criterion, corrected for small sample size (AIC_c_), and Akaike weights (AIC_w_), were used to assess the relative fit of the models [39] and the model with the highest AIC_w_ was taken as the indicated limitation type. To avoid the situation where a model was selected because certain treatments inhibited chlorophyll *a* development, i.e. chlorophyll *a* was lower than the control in those treatments, bioassay outcomes were first screened for inhibitory effects and those models and treatments removed from the candidate list, outcomes were labeled as showing +N, or +P inhibition.

Model selection was similarly used separately on the Ctrl and +NP treatments under both standard and in situ light to further classify outcomes as indicating light, co light-nutrient, or non-light limitation (Figure 3).

### Relative N vs. P Limitation and In-lake N:P Ratios

To measure the relative strength of N versus P limitation a log response ratio RR was calculated as follows:
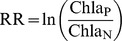
where 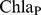 and 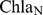 are the mean chlorophyll *a* concentrations of the three replicates at the end of the incubation in the +P and +N treatments respectively. Negative values indicate N, and positive values indicate P, as the primary limiting nutrient. RR was only calculated for experiments in which nutrient limitation was identified by the model selection (see above).

To determine the ability of in-lake N:P ratios to predict N vs. P limitation, for each N:P ratio a separate linear regression model was fit with RR as the response variable and logged in-lake TN:TP, DIN:SRP, TN:SRP and DIN:TP mass ratios as predictors. From each fitted model, the N:P ratio at which RR is predicted to be zero was used as an estimate of the ratio at which lake phytoplankton switch from being P to N limited.

Additionally, the sign of the predicted RR was used to predict outcomes as being N or P limited and a misclassification rate (MR) was calculated according to
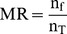
with 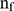 being the number of false predictions and 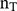 being the total number of experiments. A prediction was defined as false when from the N:P ratio the limiting nutrient was predicted to be N but RR was positive or to be P but RR was negative. Only experiments that showed nutrient limitation were used for this analysis.

All analyses were performed using R vers. 2.15.3 [40].

## Results

### Bioassays

**Figure 4:**
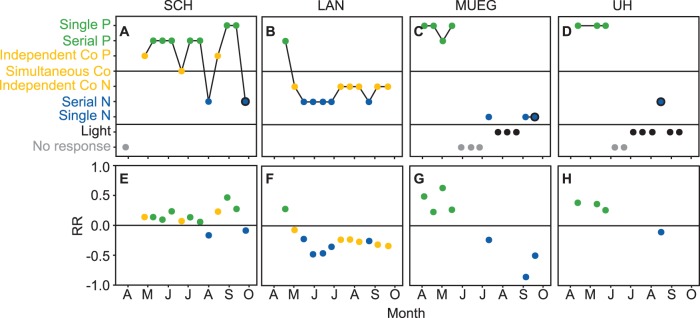
Seasonal variation of limitation types and response ratio determined by a series of bioassays (2011). A–D) Single and serial P limitation (green), independent (primary P or N) and simultaneous co-limitation (yellow), serial and single N limitation (blue), light limitation (black), co-limitation between light and nutrients (black circle around the colored point) and no response (grey). **E–H)** Response ratio (RR) indicating the relative strength of N versus P limitation. Negative values indicate N and positive values indicate P as the primary limiting nutrient. The colors are the same as those in **A–D**. RR for experiments showing no response or pure light limitation are not shown.

Nitrogen, phosphorus, and light were all at some point indicated to be the primary factor limiting phytoplankton. There was a general trend from P limitation in spring to N limitation later in the year but also differences between lakes in the relative frequency of N and P limitation (Figures 4; Table 2).

**Table 2:** Number of observations of the different limitation types in the four lakes.

	Number of Observations								
Lake	N	P	Co	Primary N	Primary P	Light	Co light/Nut.	No Response	n
SCH	2	7	3	2	9	0 (n = 4)	1 (n = 4)	1	13
LAN	5	1	6	11	1	0 (n = 0)	0 (n = 0)	0	12
MUEG	3	4	0	3	4	3 (n = 7)	1 (n = 7)	3	13
UH	1	3	0	1	3	5 (n = 9)	1 (n = 9)	2	11
Total	11	15	9	17	17	8 (n = 20)	3 (n = 20)	6	49

N: serial+single N limitation; P: serial+single P limitation; Co: independent+simultaneous co-limitation; Primary N: N+independent co-limitation (primary N); Primary P: P+independent co-limitation (primary P); Light: number of experiments showing light limitation; Co light/Nut.: number of co-limitation between light and Nutrients, n: number of experiments. In the columns Light and Co light/Nut. n gives the number of experiments where light limitation was tested.

In the deep stratified lake, SCH, the phytoplankton were predominantly limited by P (Figure 4A). From April until the end of July, P was identified as the primary limiting nutrient in 6 of 7 experiments. In late summer the limiting nutrient was more variable and switched repeatedly between P and N. Co-limitation by nutrients and light and by N and P was observed in SCH one and three times, respectively. The largest absolute value of RR, indicating the strongest limitation, was observed in September during a phase when limitation in SCH was categorized as being single P (Figure 4E).

In the shallow lake, LAN, phytoplankton were limited by P in early spring, but after a shift in early May they remained either N limited, or independent co-limited with N as the primary limiting nutrient, for the rest of the studied period (Figure 4B). The highest absolute values of RR were found in June during a period of serial N limitation (Figure 4F). In LAN only serial limitation (N and P) and no single limitation was observed.

In the temporarily stratified shallow lake, MUEG, the phytoplankton also showed a shift from P to N limitation (Figure 4C) but the period of P limitation in spring lasted longer and was followed by a period in Jun-July in which there was no response to any nutrient addition treatment. One experiment in late July indicated N limitation and then during August the phytoplankton were limited by light before returning to N limitation in September.

In the riverine lake, UH, the phytoplankton were P limited from April to the end of May, but in June they did not respond to nutrient or light addition and from July to October they were light limited with just one co-limitation between light and N (Figure 4D).

### Nutrient Concentrations, Light and Phytoplankton Biovolume

**Figure 5:**
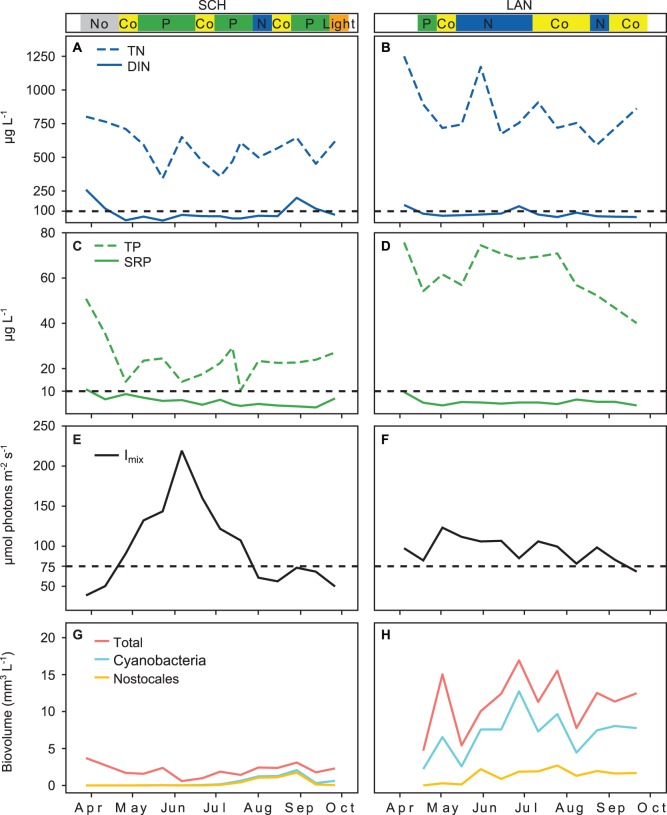
Seasonal pattern of nutrient concentration, I_mix_ and phytoplankton biovolume measured in SCH and LAN (2011). The colored bands above the graphs indicate the limitation type identified by the bioassays: where No is no limitation; Co is simultaneous or independent co-limitation; P is serial or single P limitation; N is serial or single N limitation; Light is light or co-limitation between light and nutrients. **A–D)** TN, DIN, TP and SRP; the horizontal lines mark the DIN and SRP concentrations below which N or P limitation are possible according to Maberly et al. [41]. **E** and **F)** I_mix,_ the horizontal line marks the light intensity below which in situ light treatments were conducted in the bioassays. **G** and **H)** Phytoplankton biovolume estimated according to Utermöhl [33].

**Figure 6:**
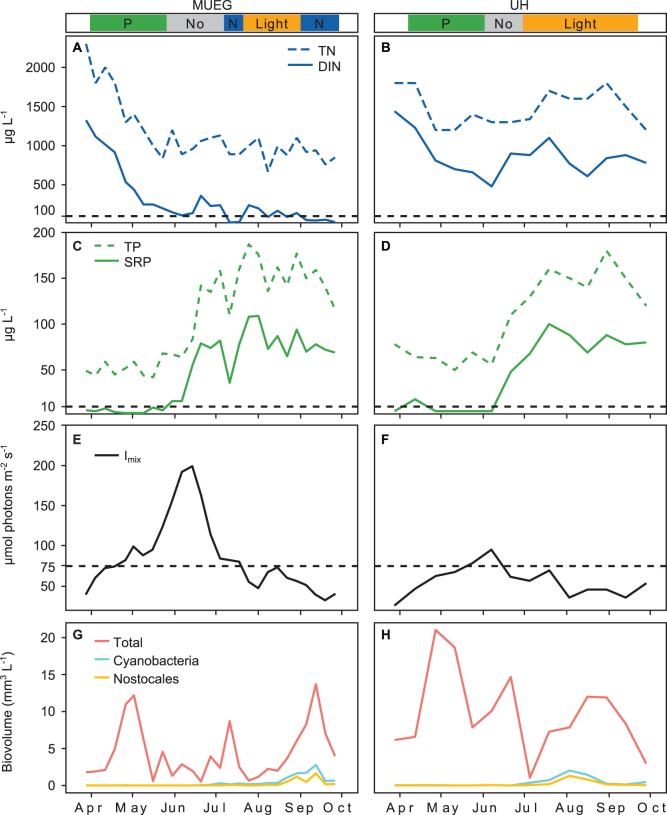
Seasonal pattern of nutrient concentration, I_mix_ and phytoplankton biovolume measured in MUEG and UH (2011). The colored bands above the graphs indicate the limitation type identified by the bioassays: where No is no limitation; Co is simultaneous or independent co-limitation; P is serial or single P limitation; N is serial or single N limitation; Light is light or co-limitation between light and nutrients. **A–D)** TN, DIN, TP and SRP; the horizontal lines mark the DIN and SRP concentrations below which N or P limitation are possible according to Maberly et al. [41]. **E** and **F)** I_mix,_ the horizontal line marks the light intensity below which in situ light treatments were conducted in the bioassays. **G** and **H)** Phytoplankton biovolume estimated according to Utermöhl [33].

The four studied lakes showed differences in their trophic status (Table 1) and in the seasonal dynamics of nutrient concentrations (Figure 5 and 6). Overall the highest nutrient concentrations were observed in UH (Figure 6B, D) and lowest in SCH (Figure 5A, C). DIN concentration in UH and MUEG was much higher than in SCH and LAN. DIN concentration in all lakes decreased in spring (Figure 5A, B and 6A, B). Both UH and MUEG showed a rapid increase of SRP and TP concentration in early summer (Figure 6C, D).

**Figure 7:**
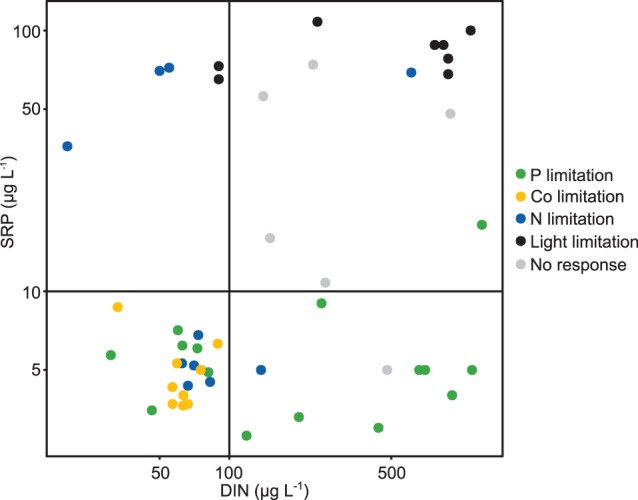
Relationships between the ambient DIN and SRP concentrations and the limitation categories. The vertical line marks the DIN concentration and the horizontal line marks the SRP concentration below which N or P limitation are possible according to Maberly et[41]. This plot shows that the results of the bioassays agree with the values given by Maberly et al. as SRP was predominantly below 10 µg L^−1^ when P limitation was observed and DIN was predominantly below 100 µg L^−1^ when N limitation was observed. Both dissolved nutrients were usually above these thresholds when light limitation or no response was observed.

SRP was very low during phases of P limitation (predominantly below 10 µg P L^−1^) and DIN was very low during phases of N limitation (predominantly below 100 µg N L^−1^) in all lakes (Figure 7). Although in SCH and LAN the dissolved forms of both nutrients were very low during the entire studied period, TP was much higher in LAN than in SCH, and SCH was predominantly P limited while LAN was N limited. The seasonal changes from P to N limitation in LAN and MUEG and from P to light limitation in UH were accompanied by a decrease of DIN in MUEG and LAN and an increase of SRP and TP in MUEG and UH. The change happened in LAN in spring and in MUEG in summer. LAN started out with lower N:P ratios than MUEG and DIN in LAN already decreased in early spring (data not shown).

The seasonal dynamic of I_mix_ is shown in Figures 5E, F and 6E, F. The highest values of I_mix_ were observed in early summer in all lakes. The threshold of I_mix_ (75 µmol photons m^−2^ s^−1^) below which an extra treatment was conducted in the nutrient enrichment bioassays was reached in SCH and MUEG in spring and late summer. In UH the measured I_mix_ was below 75 µmol photons m^−2^ s^−1^ on almost all sampling days. Light limitation was only observed when both DIN and SRP were close to or above 100 and 10 µg L^−1^ respectively (Figure 7).

The seasonal dynamic of total phytoplankton, cyanobacteria and nostocalean cyanobacteria biovolume is shown in Figures 5G, H and 6G, H. In all lakes Nostocales occurred predominantly during phases of N limitation. In SCH, MUEG and UH Nostocales, and cyanobacteria in general, occurred only in late summer, while they were observed in LAN during the whole studied period. The highest absolute biovolumes of Nostocales were observed in LAN, but the highest relative biovolume was observed in SCH in late summer.

### Prediction of the Limiting Nutrient by N:P Ratios

**Figure 8:**
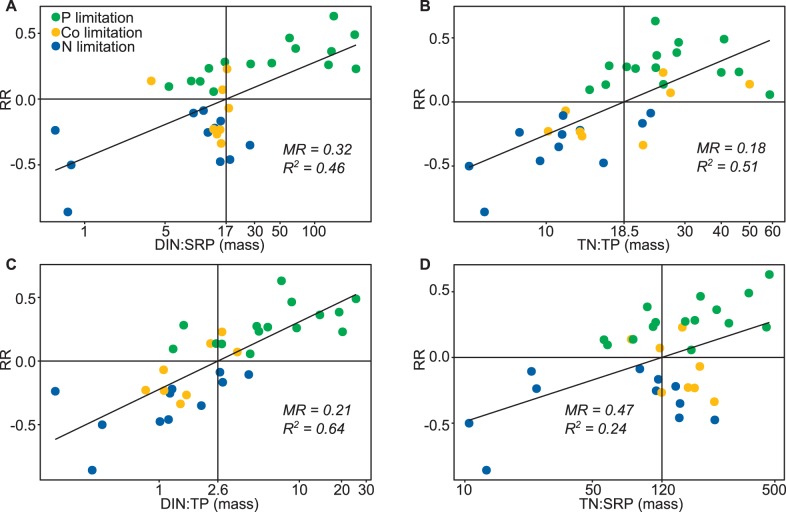
Relationships between the ambient N:P ratios and the response ratio observed in the bioassays. **A)** DIN:SRP, **B)** TN:TP, **C)** DIN:TP and **D)** TN:SRP. A positive response ratio (RR) indicates P limitation and a negative N limitation. The point at which the fitted line crosses RR = 0 identifies the ratio at which phytoplankton switch from being N to P limited. MR: Misclassification rate, *R^2^*: Coefficient of determination of the linear regression. Experiments showing no nutrient limitation were excluded.

The relationships between the in-lake DIN:SRP, TN:TP, TN:SRP and DIN:TP ratios and the P vs. N response ratio RR are shown in Figure 8. All four ratios have significant positive relationships with RR, such that high N:P ratios were associated with P, and low N:P ratios with N limitation, but *R^2^* values for the DIN:TP ratio were higher than that for the TN:TP, DIN:SRP and TN:SRP ratios (Figure 8).

Negative and positive values of RR indicate N and P respectively as the primary limiting nutrient. For each ratio, the position where the linear fit crosses the horizontal line at RR = 0 indicates the value of that ratio at which the phytoplankton are predicted to switch between N and P limitation. These points were 17, 18.5, 120 and 2.6 for the DIN:SRP, TN:TP, TN:SRP and DIN:TP ratios respectively and are indicated by vertical black lines (Figure 8). The predicted limiting nutrient for experiments in the top left and bottom right quadrants would therefore be wrong if those values were used as criteria. The number of incorrect predictions were much higher for the DIN:SRP and TN:SRP than the TN:TP or DIN:TP ratios, which is reflected in their higher misclassification rates.

### N:P Ratios

**Figure 9:**
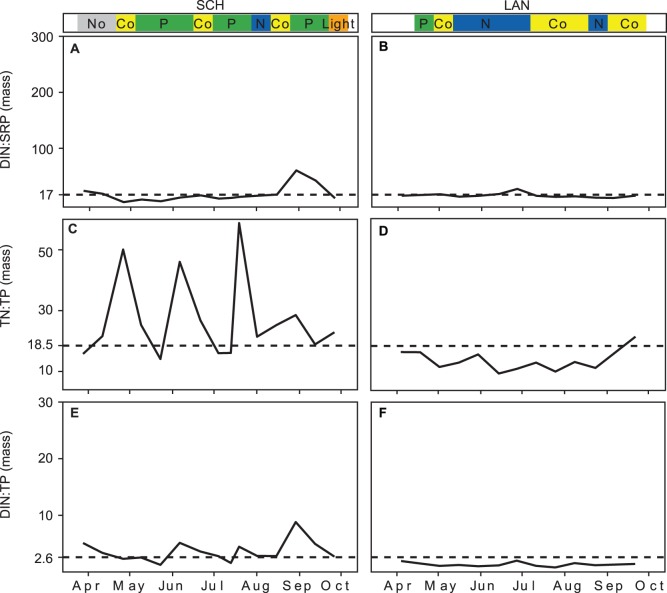
Seasonal pattern of the N:P mass ratios measured in SCH and LAN (2011). **A** and **B)** DIN:SRP, **C** and **D)** TN:TP, **E** and **F)** DIN:TP mass ratios. The colored bands above the graphs indicate the limitation type identified by the bioassays; where No is no limitation; Co is simultaneous or independent co-limitation; P is serial or single P limitation; N is serial or single N limitation; Light is light or co-limitation between light and nutrients. The horizontal lines mark the N:P ratio at which phytoplankton switched from being N to P limited based on an analysis of all four lakes combined (see Figure 8).

**Figure 10:**
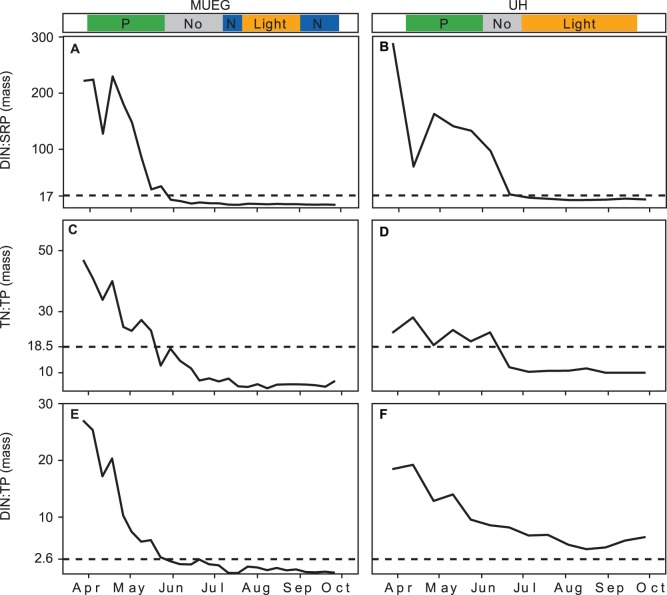
Seasonal pattern of the N:P mass ratios measured in MUEG and UH (2011). The colored bands above the graphs indicate the limitation type identified by the bioassays, where No is no limitation; Co is simultaneous or independent co-limitation; P is serial or single P limitation; N is serial or single N limitation Light is light or co-limitation between light and nutrients. The horizontal lines mark the N:P ratio at which phytoplankton switched from being N to P limited based on an analysis of all four lakes combined (see Figure 8).

The three N:P ratios TN:TP, DIN:TP and DIN:SRP were all more variable in SCH than in the other three lakes (Figure 9 A, C, E). On the two occasions in SCH when N limitation was observed all three ratios were low, although they were sometimes even lower during P limitation. In LAN the N:P ratios showed little variability (Figure 9 B, D, F); they were low during the entire studied period and were not appreciably higher on the one occasion when P limitation was observed. In MUEG and UH all three ratios showed a similar trend with very high values in spring and a sharp decrease to an extended period of low values in summer (Figure 10) during which N limitation, and co-limitation between N and light, were observed. The more eutrophic lakes LAN, MUEG and UH showed, at least in summer (MUEG and UH), lower N:P ratios and higher numbers of observed N limitation (light limitation in UH) than SCH.

## Discussion

The aim of this study was to compare the seasonal patterns of N and P limitation in four lakes of different mixing types and to test whether the limiting nutrient could be predicted from ambient nutrient concentrations and ratios.

Nutrient addition bioassays showed that the seasonal pattern of N and P limitation differed between the lakes. The deep stratified lake was predominantly limited by P, while the three shallow polymictic lakes showed a seasonal shift, with P limitation in spring and N or light limitation later in the year. These patterns of limitation matched the seasonal dynamics of nutrients and light availability, with high N:P ratios in spring and early summer and low N:P ratios and low light availability later in the year.

Ratios can only indicate the deficiency of one nutrient relative to the other; it is the absolute concentrations that determine whether nutrient limitation actually occurs. Here P limitation was observed only when SRP<10 µg L^−1^, and N limitation when DIN <100 µg L^−1^, confirming the observations of Maberly et al. [41]. However, they contrast with those of Reynolds [16], who doubted that P limitation is possible at SRP>3 µg L^−1^ and N limitation at DIN >30 µg L^−1^, as in our study N and P limitation were observed at concentrations well above these values. Nutrient affinities differ between phytoplankton species [42], [43], so differences in the phytoplankton community may explain the different findings.

When some form of nutrient limitation occurred, the primary limiting nutrient could be well predicted from ambient N:P ratios. Predictions from the DIN:TP and TN:TP ratios were more or less equally accurate and better than those from the DIN:SRP and TN:SPR ratios. This is partly in contrast to the findings of Bergström [26] and Morris and Lewis [17], where DIN:TP performed best and much better than TN:TP, but the identified values of the DIN:TP and the TN:TP mass ratios at which the phytoplankton switched from being P to N limited (2.6 and 18.5 respectively) were in good agreement with the values found in a wide range of lakes and ocean sites (see Table 3). The threshold for the TN:TP ratio we found here was higher than the Redfield ratio of 7 [24]. This is in agreement with Klausmeier et al. [44] who predict a low optimal N:P ratio of 3.7 for phytoplankton under exponential growth, but higher ratios of 16, 17 and 20 when phytoplankton are light, N, or P limited as they mostly were here. They conclude that the Redfield N:P ratio is not a universal biochemical optimum, but instead represents an average of species-specific N:P ratios.

**Table 3:** Thresholds from this study and from the literature of TN:TP and DIN:TP mass ratios that separate N and P limitation.

	TN:TP		DIN:TP			
System	N	P	N	P	*Notes	Reference
German lowland lakes	<18.5	>18.5	<2.6	>2.6		This study
American mountain lakes	<15	>25	<0.5	>4	a	[17]
Several lake and ocean sites	<9	>22.6	–	–	b	[55]
American+Swedish mountain lakes	<28	>28	<2.2	>2.2		[26]
Baltic sea	<45	>55	<2	>5	c	[27]

*a) ratios were taken from Fig. 2 of [17]. b) mass ratios were calculated from the molar ratios given by Guildford and Hecky [55]. c) ratios were taken from Fig. 5 of [27].

A reduction in N:P ratios, accompanied by a shift from P to N limitation, was observed in the three studied shallow lakes. As described by Moss et al [45] this may be a general feature of lakes and is likely due to seasonal changes in the rates of denitrification, a major sink of N in lakes [46], and P release from the sediment, which can be an important internal P source [47]. Decreasing oxygen concentrations at the sediment-water interface [48] and increasing temperatures in spring and summer promote both denitrification and the release of P [49], [50]. Large increases in both TP and SRP concentration were observed in MUEG and UH during June and were likely due to release from the sediment, which has been documented previously in MUEG [30] and in other parts of the UH river system [51]. There was no obvious increase in phytoplankton biovolume following these summer P increases. However, although their N:P ratios declined into the range where N limitation might be expected, absolute DIN concentrations remained high, particularly in UH. MUEG showed only occasional N limitation, while UH was predominantly limited by light, and therefore N limitation cannot be wholly credited for the lack of a response in biovolume.

The fact that MUEG and UH are deeper compared to LAN leads to a lower average light availability in the completely mixed water and there was frequent light limitation in MUEG and UH. Nevertheless the bioassays for these lakes conducted under standard light intensity showed a bigger response to +N treatment then to +P. So with more light available they would have been limited by N. Furthermore in the studied polymictic lakes the phytoplankton could not profit from P release from the sediment as it was limited by light or nitrogen at that time.

In the deep stratified lake SCH no clear seasonal shift in limitation was observed and this is likely explained by the isolating effect of stratification. While in shallow lakes P released from the sediment is mixed into the entire water column, during stratification of a deep lake the released P is trapped in the hypolimnion and is largely unavailable to the phytoplankton. Similarly, denitrification at the sediment-water interface is isolated from the epilimnion during stratification. Denitrification rates may also be higher in shallow lakes due to overall higher temperatures and a larger relative surface area of sediment compared with deep lakes [52].

N limited LAN and P limited SCH both had low SRP concentrations; only TP was higher in LAN than in SCH. “Luxury uptake” may explain why LAN was not P limited despite its low SRP concentrations. Many phytoplankton species are able to take up P faster than it is deployed and with this intracellular storage they are able to sustain up to four cell doublings without new P input [16].

A potential weakness of this study is that light limitation was tested only when I_mix_ was below 75 µmol photons m^−2^ s^−1^ with extra treatments under an in situ (I_mix_) in addition to the standard light intensity of 100 µmol photons m^−2^ s^−1^ (20 experiments). In other cases experiments were performed only under the standard light intensity of 100 µmol photons m^−2^ s^−1^ (29 experiments).

In 11 of the 29 experiments where light limitation was not tested, the phytoplankton were incubated at a higher light intensity than in situ (I_mix_). In these cases the phytoplankton were classified as being nutrient limited (because there was a response to at least one of the nutrient treatments) but in fact may have been either co limited by light, or indeed exclusively limited by light. However, exclusive light limitation on these occasions seems unlikely as the ambient dissolved nutrient concentrations were very low (DIN <100 µg L^−1^ and/or SRP<10 µg L^−1^).

In a further 18 of the 29 experiments where light limitation was not tested, the standard light intensity was either equal to, but in most cases lower than in situ I_mix_. In all but 3 of these phytoplankton were classified as nutrient limited when they may more correctly have been classified as co limited by light and nutrients. As they already showed a response to nutrients under 100 µmol photons m^−2^ s^−1^ a classification of exclusive light limitation would not occur even if they were to show a reaction to a higher light intensity.

In the remaining 3 experiments, where light limitation was not tested and in situ I_mix_ was higher than the standard incubation intensity, phytoplankton were classified as limited by neither light nor nutrients. Under higher light intensities the phytoplankton may have shown a response to nutrients but the high ambient concentrations of dissolved nutrients do not support this idea (DIN >100 µg L^−1^ and SRP>10 µg L^−1^). In summary, while the frequency of co-limitation by light may have been underestimated, the relative frequency of N vs. P limitation should be correct, and a greater frequency of exclusive light limitation is unlikely given the ambient nutrient concentrations and light intensities.

In the studied lakes, nostocalean cyanobacteria reached their highest biovolume in the predominantly N limited LAN, where they may have an advantage due to their ability to fix atmospheric N_2_ [53]. Unexpectedly, the highest relative abundance of nostocalean cyanobacteria was found in SCH in late summer. This might have been triggered by the short periods of N and co limitation that were observed in SCH but still it shows that nostocalean cyanobacteria can reach high relative abundances in lakes predominantly limited by P [54].

In order for water managers to best allocate resources it may be useful to know which nutrient limits phytoplankton in which lake and when. This study has shown that the frequency of nitrogen and phosphorus limitation varies between lakes and with the season and that the limiting nutrient is predictable. This study has shown that nitrogen limitation is frequent and persistent especially in shallow lakes. However it will be vital to determine whether phytoplankton biovolume can indeed be controlled by limiting the N supply and that nostocalean cyanobacteria cannot compensate by fixing N_2_ when P is plentiful as this is still controversially discussed in literature [11], [14], [15].

## Supporting Information

Table S1 **Monitoring data for the four studied lakes.** Layer (e = just the epilimnion was sapmled; whole water column was sampled); SRP_µgL (Soluble reactive phosphorus in µg P L^−1^); NOtotal_N_µGL (Nitrate+Nitrite in µg N L^−1^); NH4_N_µgL (Ammonia in µg N L^−1^); DIN_µgL (NOtotal_N_µGL+NH4_N_µgL in µg N L^−1^); TP_µgL (Total phosphorus in µg P L^−1^); TN_µgL (Total nitrogen in µg N L^−1^); DIN_SRP (DIN:SRP mass ratio); DIN_TP (DIN:TP mass ratio); TN_TP (TN:TP mass ratio); Imix (average light intensity integrated over the mixing depth in µmol photons s^−1^ m^−2^); Biovolume_total (Total phytoplankton biovolume in mm^3^ L^−1^); Biovolume_cyanobacteria (Cyanobacteria biovolume in mm^3^ L^−1^); Biovolume_nostocales (Nostocales biovolume in mm^3^ L^−1^).
(CSV)

Table S2 **Chlorophyll** ***a*** **concentrations at the end of the bioassays.** Treatment_nutrients (Ctrl = no nutrient addition; N = addition of 500 µg N L^−1^ in the form of each 250 µg N L^−1^ (NH4)2SO4 and NaNO3; P = addition of 200 µg P L^−1^ in the form of KH2PO4; NP = addition of both nutrients like in the single nutrient additions); Treatment_light (Standard light = 100 µmol photons s^−1^ m^−2^; In situ light = average Imix - for deatils see material and methods); Chlorophyll_a (Chlorophyll a concentration at the end of the experiment in µg L^−1^).
(CSV)

Table S3 **Response ratio for each experiment that was classified as nutrient limitation.** Chlrophyll_a_(N) (Chlorophyll a concentration at the end of the experiment in the +N Treatment); Chlrophyll_a_(P) (Chlorophyll a concentration at the end of the experiment in the +P Treatment); RR (Response ratio: RR = ln(Chlrophyll_a_(P) Chlrophyll_a_(N) ^−1^); RR was only calculated for experiments in which nutrient limitation was identified by the model selection).
(CSV)

## References

[1] Carpenter SR, Caraco NF, Correll DL, Howarth RW, Sharpley AN, et al (1998) Nonpoint pollution of surface waters with phosphorus and nitrogen. Ecol Appl 8: 559–568 10.1890/1051-0761(1998)0080559:NPOSWW2.0.CO2

[2] Smith VH (2003) Eutrophication of freshwater and coastal marine ecosystems a global problem. Environ Sci Pollut Res 10: 126–139 10.1065/espr2002.12.142 12729046

[3] Schindler DW, Hecky RE, Findlay DL, Stainton MP, Parker BR, et al (2008) Eutrophication of lakes cannot be controlled by reducing nitrogen input: results of a 37-year whole-ecosystem experiment. Proc Natl Acad Sci 105: 11254–11258 10.1073/pnas.0805108105 18667696 PMC2491484

[4] Lewis WM, Wurtsbaugh WA (2008) Control of lacustrine phytoplankton by nutrients: erosion of the phosphorus paradigm. Int Rev Hydrobiol 93: 446–465 10.1002/iroh.200811065

[5] Sterner RW (2008) On the phosphorus limitation paradigm for lakes. Int Rev Hydrobiol 93: 433–445 10.1002/iroh.200811068

[6] Dillon PJ, Rigler FH (1974) The phosphorus-chlorophyll relationship in lakes. Limnol Oceanogr 19: 767–773 10.4319/lo.1974.19.5.0767

[7] Schindler D (1977) Evolution of phosphorus limitation in lakes. Science 195: 260–262 10.1126/science.195.4275.260 17787798

[8] Elser JJ, Marzolf ER, Goldman CR (1990) Phosphorus and nitrogen limitation of phytoplankton growth in the freshwaters of North America: A review and critique of experimental enrichments. Can J Fish Aquat Sci 47: 1468–1477 10.1139/f90-165

[9] Elser JJ, Bracken MES, Cleland EE, Gruner DS, Harpole WS, et al (2007) Global analysis of nitrogen and phosphorus limitation of primary producers in freshwater, marine and terrestrial ecosystems. Ecol Lett 10: 1135–1142 10.1111/j.1461-0248.2007.01113.x 17922835

[10] Downing JA, McCauley E (1992) The nitrogen: phosphorus relationship in lakes. Limnol Oceanogr 37: 936–945 10.4319/lo.1992.37.5.0936

[11] Schindler DW (2012) The dilemma of controlling cultural eutrophication of lakes. Proc R Soc B Biol Sci 279: 4322–4333 10.1098/rspb.2012.1032 PMC3479793 22915669

[12] Spivak AC, Vanni MJ, Mette EM (2011) Moving on up: can results from simple aquatic mesocosm experiments be applied across broad spatial scales? Freshw Biol 56: 279–291 10.1111/j.1365-2427.2010.02495.x

[13] Lewis, Jr WM, Wurtsbaugh WA, Paerl HW (2011) Rationale for control of anthropogenic nitrogen and phosphorus to reduce eutrophication of inland waters. Environ Sci Technol. 10.1021/es202401p 22070635

[14] Scott JT, McCarthy MJ (2010) Nitrogen fixation may not balance the nitrogen pool in lakes over timescales relevant to eutrophication management. Limnol Ocean 55: 1265–1270 10.4319/lo.2010.55.3.1265

[15] Paterson MJ, Schindler DW, Hecky RE, Findlay DL, Rondeau KJ (2011) Comment: Lake 227 shows clearly that controlling inputs of nitrogen will not reduce or prevent eutrophication of lakes. Limnol Oceanogr 56: 1545–1547 10.4319/lo.2011.56.4.1545

[16] Reynolds CS (2006) Ecology of phytoplankton. Cambridge; New York: Cambridge University Press.

[17] Morris DP, Lewis WM (1988) Phytoplankton nutrient limitation in Colorado mountain lakes. Freshw Biol 20: 315–327 10.1111/j.1365-2427.1988.tb00457.x

[18] Butt AJ, Brown BL (2000) The cost of nutrient reduction: A case study of Chesapeake Bay. Coast Manag 28: 175–185 10.1080/089207500263585

[19] Rabotyagov S, Campbell T, Jha M, Gassman PW, Arnold J, et al (2009) Least-cost control of agricultural nutrient contributions to the Gulf of Mexico hypoxic zone. Ecol Appl 20: 1542–1555 10.1890/08-0680.1 20945758

[20] Levine SN, Schindler DW (1999) Influence of nitrogen to phosphorus supply ratios and physicochemical conditions on cyanobacteria and phytoplankton species composition in the Experimental Lakes Area. Can J Fish Aquat Sci 56: 451–466 10.1139/f98-183

[21] Sommer U (1989) Nutrient status and nutrient competition of phytoplankton in a shallow, hypertrophic lake. Limnol Oceanogr 34: 1162–1173 10.4319/lo.1989.34.7.1162

[22] Holmgren SK (1984) Experimental lake fertilization in the Kuokkel area, northern Sweden. Phytoplankton biomass and algal composition in natural and fertilized subarctic lakes. Int Rev Gesamten Hydrobiol Hydrogr 69: 781–817 10.1002/iroh.19840690603

[23] Hecky RE, Kilham P (1988) Nutrient limitation of phytoplankton in freshwater and marine environments: A review of recent evidence on the effects of enrichment. Limnol Oceanogr 33: 796–822 10.4319/lo.1988.33.4part2.0796

[24] Redfield AC (1958) The biological control of chemical factors in the environment. Am Sci 46: 205–221. 24545739

[25] Sterner RW, Andersen T, Elser JJ, Hessen DO, Hood JM, et al (2008) Scale-dependent carbon: nitrogen: phosphorus seston stoichiometry in marine and freshwaters. Limnol Oceanogr 53: 1169–1180 10.4319/lo.2008.53.3.1169

[26] Bergström A-K (2010) The use of TN:TP and DIN:TP ratios as indicators for phytoplankton nutrient limitation in oligotrophic lakes affected by N deposition. Aquat Sci 72: 277–281 10.1007/s00027-010-0132-0

[27] Ptacnik R, Andersen T, Tamminen T (2010) Performance of the Redfield Ratio and a family of nutrient limitation indicators as thresholds for phytoplankton N vs. P limitation. Ecosystems 13: 1201–1214 10.1007/s10021-010-9380-z

[28] Grüneberg B, Rücker J, Nixdorf B, Behrendt H (2011) Dilemma of non-steady state in lakes - development and predictability of in-lake P concentration in dimictic lake Scharmützelsee (Germany) after abrupt load reduction. Int Rev Hydrobiol 96: 599–621 10.1002/iroh.201111287

[29] Nixdorf B, Deneke R (1997) Why ‘very shallow’ lakes are more successful opposing reduced nutrient loads. Dev Hydrobiol 119: 269–284 10.1007/978-94-011-5648-628

[30] Köhler J, Hilt S, Adrian R, Nicklisch A, Kozerski HP, et al (2005) Long-term response of a shallow, moderately flushed lake to reduced external phosphorus and nitrogen loading. Freshw Biol 50: 1639–1650 10.1111/j.1365-2427.2005.01430.x

[31] Knösche R (2006) Organic sediment nutrient concentrations and their relationship with the hydrological connectivity of floodplain waters (River Havel, NE Germany). Hydrobiologia 560: 63–76 10.1007/s10750-005-0983-x

[32] DEV (1960) Deutsche Einheitsverfahren zur Wasser-, Abwasser- und Schlamm-Untersuchung. Weinheim: Wiley-VCH Verlag GmbH.

[33] Utermöhl H (1958) Zur Vervollkommnung der quantitativen Phytoplankton-Methodik. Mitt Int Ver Theor Angew Limnol 9: 1–38.

[34] Wiedner C, Rücker J, Brüggemann R, Nixdorf B (2007) Climate change affects timing and size of populations of an invasive cyanobacterium in temperate regions. Oecologia 152: 473–484 10.1007/s00442-007-0683-5 17375336

[35] Hilt S, Henschke I, Rücker J, Nixdorf B (2010) Can submerged macrophytes influence turbidity and trophic state in deep lakes? Suggestions from a case study. J Environ Qual 39: 725–733 10.2134/jeq2009.0122 20176845

[36] Kirk JTO (2011) Light and photosynthesis in aquatic ecosystems. Cambridge, UK; New York: Cambridge University Press.

[37] Harpole WS, Ngai JT, Cleland EE, Seabloom EW, Borer ET, et al (2011) Nutrient co-limitation of primary producer communities. Ecol Lett Online 14: 852–862 10.1111/j.1461-0248.2011.01651.x 21749598

[38] Andersen T, Saloranta TM, Tamminen T (2007) A statistical procedure for unsupervised classification of nutrient limitation bioassay experiments with natural phytoplankton communities. Limnol Ocean Methods 5: 111–118 10.4319/lom.2007.5.111

[39] Burnham KP, Anderson DR (2002) Model selection and multimodel inference: A Practical Information-Theoretic Approach. Springer.

[40] R Core Team (2012) R: A language and environment for statistical computing. Vienna, Austria. Available: http://www.R-project.org/.

[41] Maberly SC, King L, Dent MM, Jones RI, Gibson CE (2002) Nutrient limitation of phytoplankton and periphyton growth in upland lakes. Freshw Biol 47: 2136–2152 10.1046/j.1365-2427.2002.00962.x

[42] Gotham IJ, Rhee G-Y (1981) Comparative konetic studies of nitrate-limited growth and nitrate uptake in phytoplankton in continuous culture. J Phycol 17: 309–314 10.1111/j.1529-8817.1981.tb00856.x

[43] Gotham IJ, Rhee G-Y (1981) Comparative konetic studies of phosphate-limited growth and phosphate uptake in phytoplankton in continuous culture. J Phycol 17: 257–265 10.1111/j.1529-8817.1981.tb00848.x

[44] Klausmeier CA, Litchman E, Daufresne T, Levin SA (2004) Optimal nitrogen-to-phosphorus stoichiometry of phytoplankton. Nature 429: 171–174 10.1038/nature02454 15141209

[45] Moss B, Jeppesen E, Søndergaard M, Lauridsen TL, Liu Z (2012) Nitrogen, macrophytes, shallow lakes and nutrient limitation: resolution of a current controversy? Hydrobiologia 710: 3–21 10.1007/s10750-012-1033-0

[46] Lijklema L (1994) Nutrient dynamics in shallow lakes: effects of changes in loading and role of sediment-water interactions. Hydrobiologia 275–276: 335–348 10.1007/BF00026724

[47] Hupfer M, Lewandowski J (2008) Oxygen controls the phosphorus release from lake sediments - a Long-lasting paradigm in limnology. Int Rev Hydrobiol 93: 415–432 10.1002/iroh.200711054

[48] Wetzel RG (2001) Limnology: lake and river ecosystems. 3rd ed. San Diego: Academic Press. 1006 p.

[49] Jensen HS, Andersen FO (1992) Importance of temperature, nitrate, and pH for phosphate release from aerobic sediments of four shallow, eutrophic lakes. Limnol Oceanogr 37: 577–589 10.4319/lo.1992.37.3.0577

[50] Veraart AJ, de Klein JJM, Scheffer M (2011) Warming can boost denitrification disproportionately due to altered oxygen dynamics. PLoS ONE 6: e18508 10.1371/journal.pone.0018508 21483809 PMC3069112

[51] Schauser I, Chorus I (2009) Water and phosphorus mass balance of Lake Tegel and Schlachtensee – A modelling approach. Water Res 43: 1788–1800 10.1016/j.watres.2009.01.007 19232667

[52] Scheffer M (2004) Ecology of shallow lakes. Dordrecht; Boston: Kluwer Academic Publishers.

[53] Smith VH, Bennet SJ (1999) Nitrogen: phosphorus supply ratios and phytoplankton community structure in lakes. Arch Für Hydrobiol 146: 37–53.

[54] Dolman AM, Rücker J, Pick FR, Fastner J, Rohrlack T, et al (2012) Cyanobacteria and cyanotoxins: The influence of nitrogen versus phosphorus. PLoS ONE 7: e38757 10.1371/journal.pone.0038757 22719937 PMC3376147

[55] Guildford S, Hecky R (2000) Total nitrogen, total phosphorus, and nutrient limitation in lakes and oceans: Is there a common relationship? Limnol Oceanogr 45: 1213–1223 10.4319/lo.2000.45.6.1213

